# Unveiling the Enigma: A Rare Case of a Massive Gartner’s Cyst Inducing Acute Urinary Retention

**DOI:** 10.7759/cureus.52476

**Published:** 2024-01-18

**Authors:** Aishwarya Gupta, Neema Acharya, Sandhya Pajai, Preeti Mishra, Jalormy S Joshi

**Affiliations:** 1 Department of Obstetrics and Gynaecology, Jawaharlal Nehru Medical College, Datta Meghe Institute of Higher Education and Research, Wardha, IND; 2 Department of Pathology, Jawaharlal Nehru Medical College, Datta Meghe Institute of Higher Education and Research, Wardha, IND

**Keywords:** wolffian remnants, mullerian cysts, vaginal cysts, acute urinary retention, gartner's cyst

## Abstract

Vaginal cysts can occur due to embryonic remnants, misplaced tissue, or an abnormality in the urinary system. They are a common occurrence and usually indicate non-cancerous conditions. A case is presented here of a 35-year-old female para three living three who reported to the emergency room with complaints of acute retention of urine with something coming out of her vagina over the last two years. She was managed operatively by vaginal cystectomy, which led to the resolution of the symptoms.

## Introduction

In both males and females, the urogenital system contains two types of ducts, the Wolffian and the Müllerian, which are essential for the reproductive and urinary systems. In females, the Müllerian ducts unite during the eighth week of embryonic development to form the uterus, cervix, and upper vagina. Additionally, during female fetal development, the Wolffian ducts typically regress. However, if they remain vestigial, Gartner's duct cysts may form [1]. Vaginal cysts are relatively uncommon, but their histology has allowed for the identification and classification of various types based on the composition of the cyst wall's epidermis. Mullerian cysts, endometroid cysts, epidermal inclusion cysts, Bartholin cysts or abscesses, Gartner's duct cysts, and unidentified variants are among these types [2].

Mullerian cysts are typically tiny, measuring between 0.1 and 2 cm. They may occasionally get larger and are often misdiagnosed as different conditions, such as uterovaginal prolapse, cystocele, rectocele, or urethral diverticulum [3]. True Gartner's duct cysts are most commonly seen along the anterolateral walls of the proximate vagina. Bartholin cysts are most commonly seen on the posterolateral walls of the distant vagina, near the labia majora. Gartner's duct cysts often turn out to be tiny and asymptomatic, with a diameter of less than 2 cm. As the cysts grow, they may be confused with other medical conditions, including urethral diverticulum or cystocele. The largest Gartner duct cyst discovered to date was 16 cm by 15 cm by 8 cm in size. Larger cysts may obstruct vaginal delivery or cause dyspareunia [4].

Cysts in the Skene glands can be found beneath the perineal membrane, near the anterolateral urethral orifice. Bartholin gland cysts are situated below the perineal membrane, adjacent to the posterolateral area of the vaginal introitus. Gartner duct cysts typically develop above the perineal membrane, originating from the anterolateral wall of the upper vagina [3].

Lower urinary tract symptoms (LUTS) comprise seven categories: storage, voiding, postmicturition, sexual function, pelvic organ prolapse, lower urinary tract discomfort, and lower urinary tract dysfunction syndrome. As many as 26% of women aged 40 and over may experience symptoms of the lower urinary tract, which is a group of non-organ-specific symptoms that can worsen with age [5]. The incidence of urine retention in women is extremely low, averaging 0.07 cases per 1000 people annually [6].

Gartner's duct cysts are typically not associated with urine retention. However, a large or abnormal remnant can put pressure on neighboring structures like the urethra or bladder neck, leading to urinary flow obstruction and retention. Understanding the varied diagnosis of benign cystic vaginal lesions and accompanying anomalies will assist in examination and therapy.

## Case presentation

A 35-year-old para-three-living-three woman with all previous cesarean deliveries with last childbirth three years ago came to the hospital outpatient department complaining of acute retention of urine for four days and something coming out of her vagina for two years. Her symptoms started slowly and gradually escalated. There was no evidence of swelling growing or increasing in size due to exertion or lifting of heavy weights. She had regular menstrual cycles of 28-30 days with three to four days of flow, not associated with dysmenorrhea or the passage of clots. Except for mild discomfort during sexual intercourse, she had no history of discharge from the vagina, fever, injury, backache, or stomach or pelvic pain.

**Figure 1:**
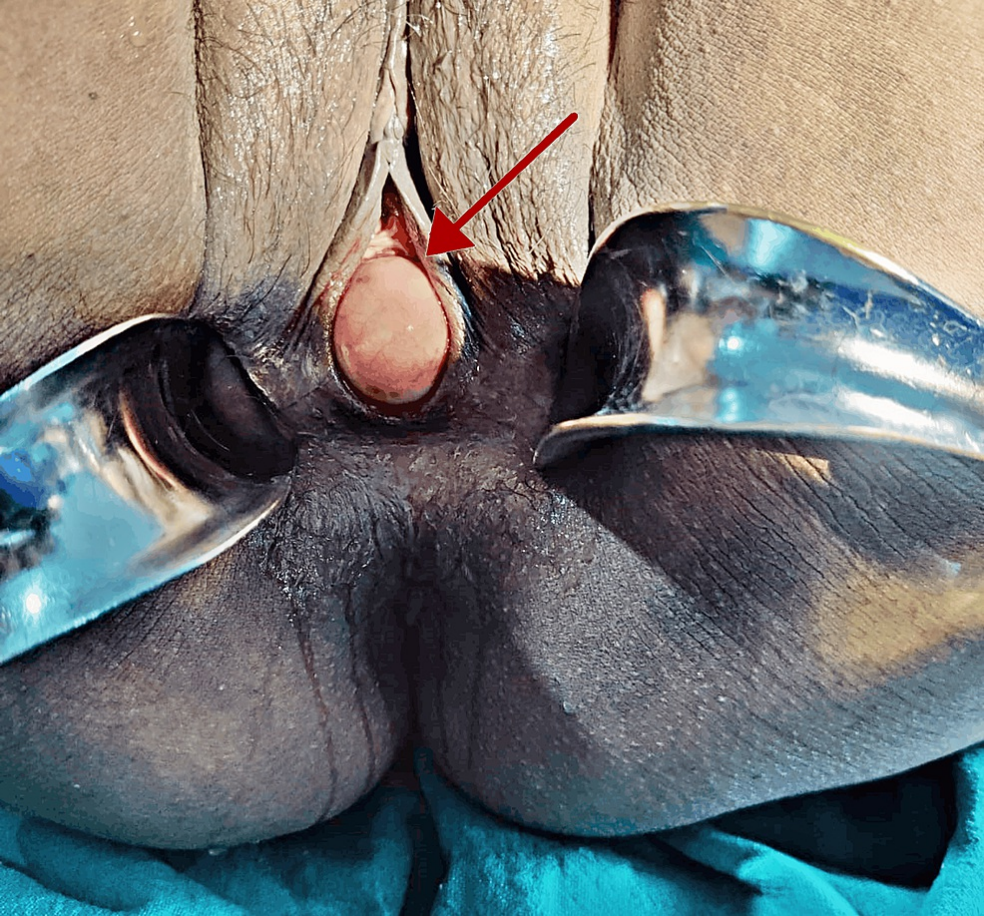
On speculum examination, a non-tender cystic enlargement seen covered by the vaginal mucosa extending from the vagina.

On initial examination, the patient appeared to be cognizant and well-oriented to time, place, location, and person. During the abdominal examination, the abdomen felt soft and painless, with a visible scar from a prior lower-section cesarean procedure. There were no detectable lumps or enlargement of internal organs. External genitalia were found to be normal on a local inspection. Upon speculum inspection, a tense, cystic growth measuring 6 cm x 4 cm x 3.5 cm and covered by the vaginal mucosa seemed to be extending from the vagina, suggesting that the swelling was coming from the anterior vaginal wall. There was no cough reflex, and no vaginal rugosities were seen, as shown in Figure 1. Upon vaginal examination, the meatus of the urethra was the lowest limit of the cyst, and its greatest extent was 1 cm below the upper lip of the cervix. The uterus was normal in size, non-tender, and mobile, having no forniceal tenderness.

Investigations revealed that the blood biochemistry was mostly normal, which is summarized in Table 1.

**Table 1:** Laboratory parameters of the patient with reference range.

S. NO.	Investigations	Observed value	Expected value
1	Haemoglobin (gm%)	12.2	12-16
2	WBCs (cu mm)	10300	4000-11000
3	Glycated haemoglobin (HBA1c)	4.8	≤ 5.6
4	Serum urea (mg/dL)	20	6-24
5	Serum creatinine (mg/dL)	0.9	0.7-1.2
6	Serum sodium (mEq/L)	138	131-145
7	Serum potassium (mmol/L)	4.1	3.6-5.2
8	Thyroid-stimulating hormone (mlU/L)	2.6	0.5-5.0
9	Free T3 (pg/mL)	3.12	2.3-4.1
10	Free T4 (pg/mL)	11.16	9.0-17.0
11	Albumin	3.8 g/dl	3.5-5.0 g/dl
12	Aspartate aminotransferase	40 U/L	<50 U/L
13	Alanine aminotransferase	28 U/L	17-59 U/L
14	Total bilirubin	0.8 mg/dl	0.2-1.3 mg/dl
15	International normalized ratio	0.9	0.8-1.1
16	Prothrombin time	11.7	11.9
17	Activated partial thromboplastin time (APTT)	30.2	29.5

**Figure 2:**
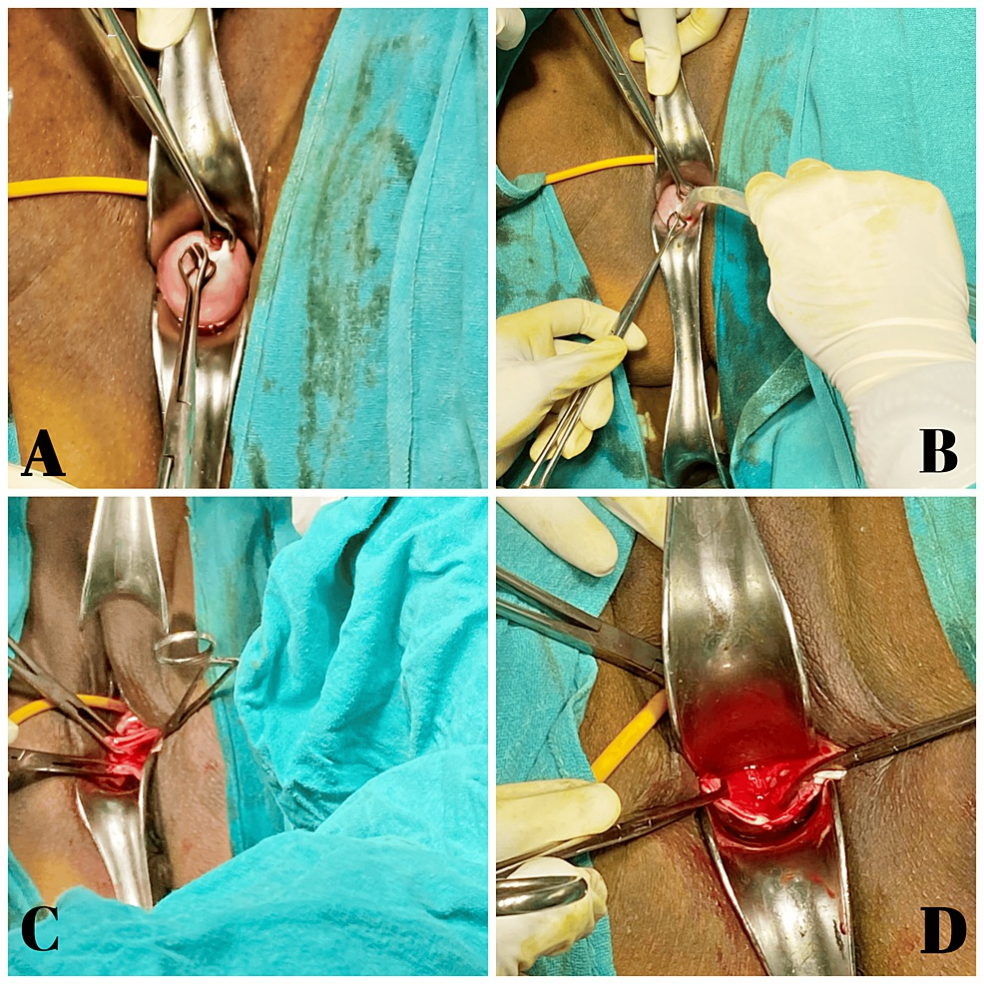
Intraoperative findings. (A) After visualization of the cyst, it is held with Babcock's forceps; (B) a small incision is made on the cyst, and the contents of the cyst are aspirated; (C) the cyst is opened using blunt and sharp dissections; (D) excess vaginal tissue removed and mucosa sutured with absorbable sutures.

When ultrasound was performed, it revealed a big, inflated bladder with 300 ml of post-void residual urine and a cystic lesion measuring 3.8 cm × 3.5 cm in the vaginal wall. The patient was slated for a vaginal cystectomy under spinal anaesthesia. Following the patient's preparation through cleaning, painting, and draping, a catheter was inserted as a precautionary measure to avert inadvertent urethral damage, leading to a notable decrease in the cyst's size. On the anterior vaginal wall, a minor vertical incision was made. Blunt as well as sharp dissection techniques were employed to remove the cyst. Precautions were taken to avoid urethral and bladder damage. A fibrous adhesion held the cyst to the vagina. Figures 2A-2D show how the mucosa was closed using absorbable sutures after the remaining vaginal tissue was excised.

**Figure 3:**
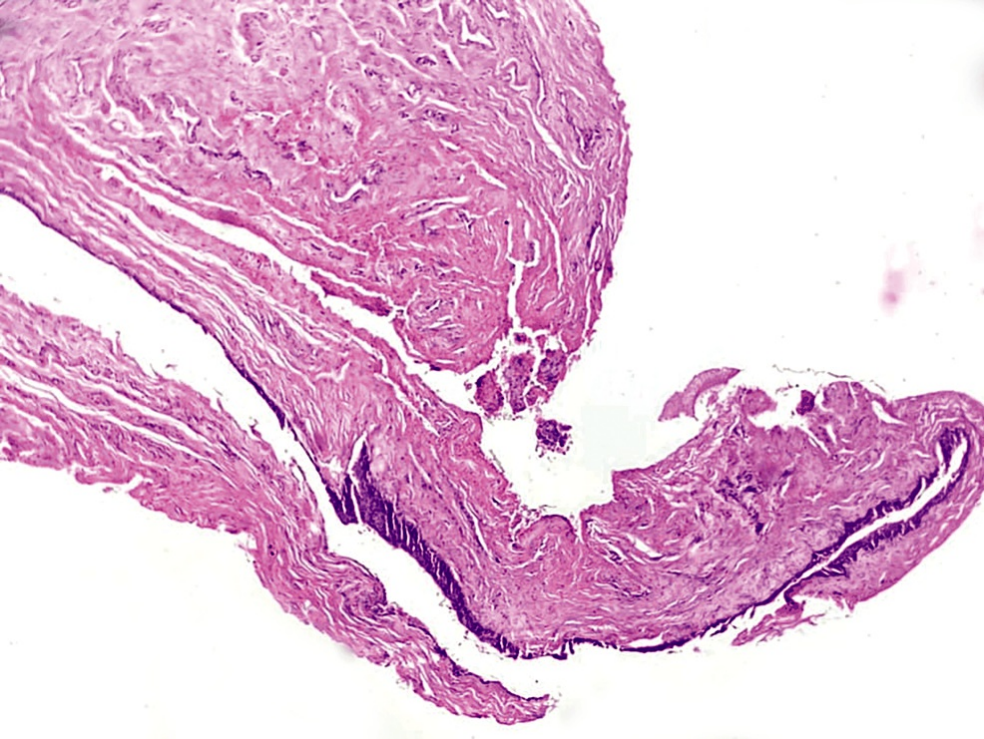
The given section was stained with haematoxylin, and the eosin stain on scanner view shows the cyst wall is lined by tall columnar epithelium. Deeper tissue shows fibro collagenous and muscle tissue. Impression: epithelial cyst-mesonephric type.

The excess vaginal mucosa and the excised cyst were sent for histopathological examination. On histopathology, the given section was stained with haematoxylin, and the eosin stain on the scanner view shows the cyst wall is lined by tall columnar epithelium. Deeper tissue shows fibro-collagenous and muscle tissue, which was suggestive of the epithelial cyst-mesonephric type (Gartner's duct cyst), as shown in Figure 3.

**Figure 4:**
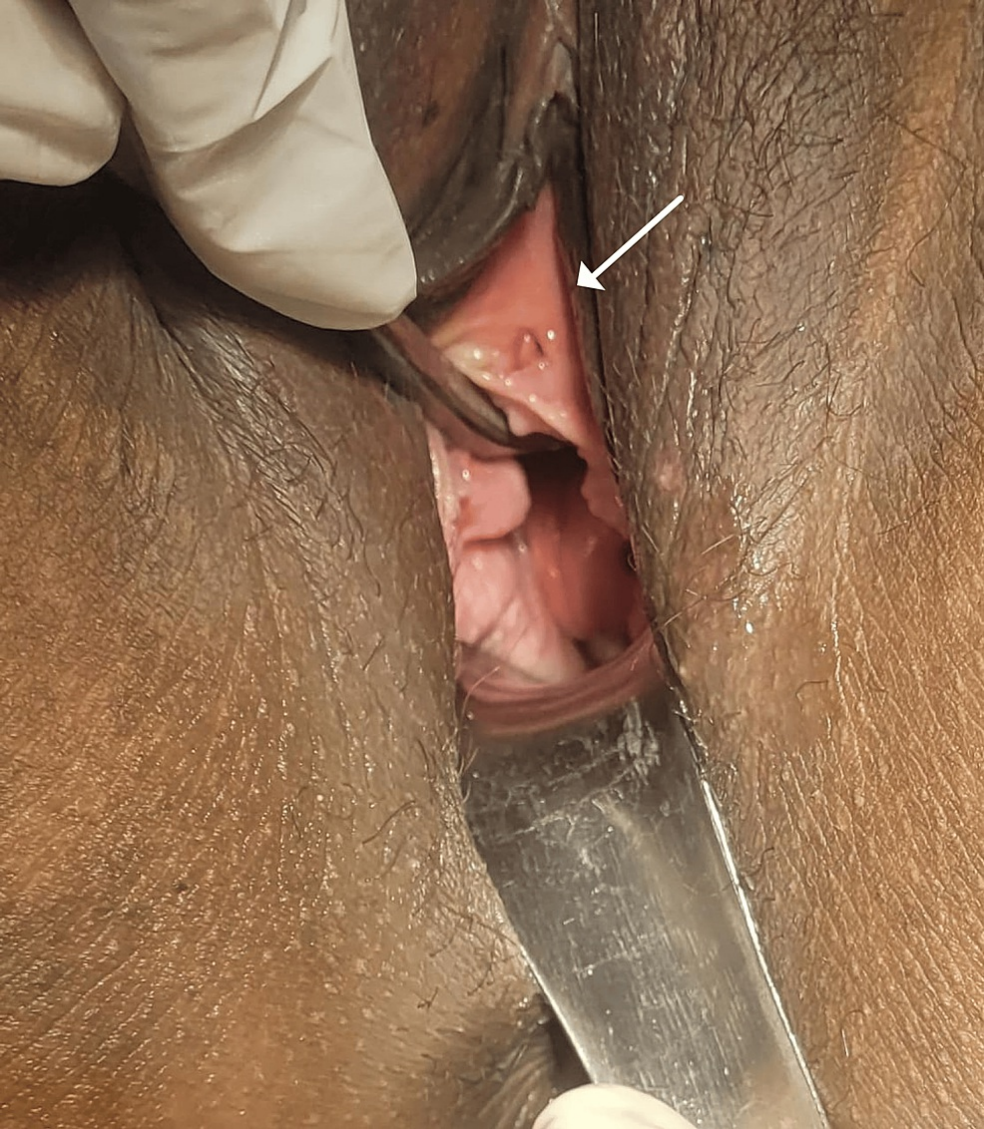
Post-cystectomy follow-up after three months.

The patient experienced a smooth recovery following the surgical procedure and was released from the hospital after a 72-hour observation period. Throughout this time, the patient was maintained with a catheter in place to ensure ongoing monitoring for any potential inadvertent damage to the urethra during the surgery. Additionally, the patient received injectable antibiotics during this period to proactively prevent the occurrence of postoperative sepsis. Two follow-up consultations were conducted, and there were no symptoms observed, as shown in Figure 4.

## Discussion

The Gartner's duct is present when the fetus begins to develop in the womb. During embryonic development, the Müllerian ducts combine to form the uterus, cervix, and upper vagina in the eighth week. Each of these structures is surrounded by a layer of glandular pseudostratified columnar epithelium. In females, Wolffian (mesonephric) ducts often regress, leaving behind the Gartner's duct, epoöphoron, and paroöphoron. If either of the ducts remains, it might gather fluid and eventually develop into a vaginal wall cyst [7]. Gartner's duct cysts typically remain silent and are diagnosed through a routine gynecologic exam. However, if they grow to a detectable size, they can cause visible skin tags, urinary retention, pressure, itching, dyspareunia, pelvic pain, or a protrusion from the vagina, necessitating surgical excision [8].

Post-void residual (PVR) urine is a measure of urine that remains in the bladder following micturition. Catheterization, or non-invasive ultrasonography, is an effective way to measure it. Because PVR differs from person to person, repeated measurements are sometimes required. There is no maximum capacity that can be regarded as normal based on research. According to the Agency for Health Care Policy and Research (AHCPR) criteria, normal bladder emptying is defined as a PVR of below fifty millilitres. A PVR of over two hundred millilitres, on the other hand, suggests inadequate emptying [9].

Usually, diagnoses for this condition are discovered unintentionally during routine pelvic examinations, and many growths have no noticeable symptoms. Diagnostic confirmation is typically done through a transvaginal ultrasound. Treatment options vary depending on the patient's symptoms and preferences. Clinical observation is often recommended for women who do not experience symptoms. Histologic examination can be used to correctly identify cellular remains originating from non-mucin-secreting low-columnar or cuboidal epithelium [10].

Although histologic analysis can be utilized to precisely identify the cellular remnants, it is not necessarily required in clinical practice. Endometriosis, prolapsed uterus, prolapsed urethra, leiomyoma, sarcoma botryoides, malignant tumours (like squamous cell carcinoma of the vagina), Skene's gland cysts or abscesses, Bartholin's gland cysts or abscesses, and ureteroceles are all possible diagnoses [8]. Surgery is seldom performed in this scenario since it is complicated and is not recommended if a patient does not have severe symptoms. When a patient has symptoms, the first course of treatment may include cyst drainage, injections, or aspiration, as well as intra-cystic tetracycline. Excision or marsupialization is recommended for big, symptomatic, or recurring cysts.

A simple and minimally invasive treatment for Gartner's duct cysts is cyst marsupialization. This procedure allows for the identification of the cysts through histological analysis while also resulting in minimal surgical scarring. It has been confirmed to be effective through long-term follow-up, with no adverse effects or recurrence. The treatment for multiloculated recurrences includes frequent surveillance, sclerotherapy, and marsupialization. A mass wall biopsy is indicated for elderly people to rule out the risk of neoplasia. It should be noted, however, that the probability of Gartner's cysts undergoing malignant transformation is exceptionally low [1].

## Conclusions

Conservative therapy is a safe choice for asymptomatic patients with Gartner's cysts, given their low incidence. However, surgery is highly advised in cases with severe symptoms or big cysts, and patients should be closely monitored after the procedure. This approach has the advantage of reducing recurrence rates. Surgical intervention is a crucial and effective approach for managing a massive Gardner's cyst inducing acute urinary retention. The choice of surgery should be based on a thorough evaluation of the patient's condition, with the aim of relieving obstruction and restoring normal urinary function.
